# The patient musician: a qualitative investigation of professional classical musicians who previously suffered from depression

**DOI:** 10.1186/s12889-025-22656-w

**Published:** 2025-05-01

**Authors:** Michaela Korte, Deniz Cerci, Roman Wehry, Renee Timmers, Victoria J. Williamson

**Affiliations:** 1 Department of Music, The University of Sheffield, Western Bank, Sheffield, S10 2TN UK; 2 Klinik für Forensische Psychiatrie, Universitätsmedizin Rostock, Zentrum für Nervenheilkunde, Gehlsheimer Straße 20, 18147 Rostock, Germany; 3 Department of Anesthesiology, Medical College of Wisconsin, 9200 W. Wisconsin Ave., Milwaukee, WI 53226 USA; 4 Independent Academic, Barcelona, Spain

**Keywords:** Depression, Musicians, Treatment options, Musician-specific care

## Abstract

**Background:**

Music students are more vulnerable to depression than their peers in many other academic fields. Contributing factors include early specialization and social isolation resulting from long hours of practice, the prevalence of chronic pain and the challenges associated with developing a coherent identity within the context of an inherently competitive field. Moreover, the culture around music seems to reward suffering at times, which may impede seeking professional medical assistance.

**Method:**

Seven professional classical musicians participated in a written semi-structured interview. This study aimed to gain insight into the experiences of classical musicians who have been medically diagnosed with depression at some point during their studies and in their subsequent careers. The objective was to identify the specific challenges faced by musicians and the (effective) strategies employed. Furthermore, the focus was on enhancing awareness of the existing resources and developing strategies for further improvement.

**Results:**

The findings indicated that all participants were embedded within a robust social support system that facilitated their ability to seek medical assistance. They received a standard medical treatment, which included cognitive-behavioral therapy with or without medication. Financial difficulties, the impact of an inability to work, and external expectations were among the most prevalent challenges. Distinctive challenges specific to musicians during depression included a decline in their creative output, a loss of voice, heightened anxiety, and difficulties to engage with emotions. Participants indicated that their strategies for enhancing their health were largely based on their individual resourcefulness and not necessarily a direct result of their formal education.

**Conclusion:**

The findings indicate the necessity of integrating a positive approach to (performance) anxiety, establishing secure environments for vulnerable students beyond their academic settings, and enhancing students’ awareness of available (medical) standard multidisciplinary treatments. The emergence of a specialized, board-certified field of musicians’ medicine within the medical field is a promising development that could provide support to musicians and facilitate their long-term career prospects.

**Supplementary Information:**

The online version contains supplementary material available at 10.1186/s12889-025-22656-w.

## Introduction

Depressive disorders remain a leading cause of disability affecting 280 million people worldwide [1]. Research has identified several at risk groups, one of which is the age group between young adolescence and pre-adulthood (17–25 years) [2, 3]. The age of onset is a critical factor as it is associated with multiple burdens in later life, ranging from high chronicity to psychopathology in families or (socio)behavioral problems to name a few [4, 5]). The current bio-psycho-socio model of depression has allowed for a better understanding of the different challenges and for timely interventions that are better tailored to the needs of each group. Early intervention has been shown to be effective and to require fewer resources than diagnosis at a later stage. These include psychotherapy (i.e. cognitive behavioral therapy (CBT)) individual or in group with or without medication depending on severity and comorbidities [6]. In this context CBT has been shown to reliably reduce relapse and recurrence of depression and facilitate recovery as demonstrated by Legemaat et al. in a twenty-year follow-up study [7].

One subgroup that has consistently shown greater vulnerability to depressive disorders are students in higher education, compared to their peers in apprenticeships or other training [8, 9]. Courses such as medicine, dentistry or music contribute to a higher risk of suffering from depression compared to other study courses [10]. What makes a music student more vulnerable to depression than some of their peers?

In the literature, early specialization and countless hours of practice, most of it before entering music college, social isolation, identity foreclosure, (difficult) relationships with teachers, burnout, injury and (chronic) pain, psychological pressure and perfectionism, tend to be seen as the reasons for the high prevalence of depression in student musicians [11, 12, 13, 14, 15, 16]. In addition to their personal sacrifice, students also face high levels of job insecurity and financial instability after graduation from music college [16, 17]. From this perspective, it becomes evident that a multitude of challenges must be overcome before one can even hope to embark on a fruitful career.

The choice of a highly specialized learning environment and (instrumental/vocal) teacher appears to be crucial for subsequent success. In these learning environments, psychological preparation should constitute an indispensable aspect of talent development. The conventional approach in music college has been on the selection of “talent” as well as relying on practice and expert instruction to enable progress. While talent has different meanings in different circumstances, as a soft term, it is important to note that it appears on music college applications, websites etc. without operationalization. However, we believe that talent in this context describes a wide variety of (potential) music students, either as “raw material” or as the product of a complex training/development process [16], a potential new student who impresses the jury with their choice of works and personal interpretation, or with their exceptional vocal abilities and qualities. This approach is inadequate for addressing the multifaceted aspects of talent development, thereby failing to provide performers with the optimal level of preparation [18]. The UK offers two ways of studying music, at music college/conservatoire, a route preferred by those aiming to attain elite music status, and university which offers a more academic approach to the subject [19]. Perhaps not surprisingly, students at music colleges have shown a significantly higher prevalence of depression than their peers who studied music at university [10]. However, despite the higher prevalence of depression, music college students also had a significantly better social network and were significantly less affected by negativity and (chronic) pain than university music students [19]. Both variables are among those that serve as protectors and/or mitigate the effects of depression. This series of findings raises the question of whether the prevailing theory of music colleges prioritizes individual-level factors in students, such as maladaptive coping strategies, or focuses on individual (struggling) students rather than also considering the root of the problem in their larger educational/organizational structure.

There is no good reason why larger organizational factors (i.e. power imbalance), social processes (e.g. conformity to dominant values), and organizational stressors (e.g. professional uncertainty) that are relevant to social and behavioral science and in sports psychology, should not also apply to music psychology/performance science [20, 21].

In addition, the culture around music rewards those who suffer from depression, as depression is said to enable musicians to reach a special peak of creativity [22, 23]. This issue is further complicated by the fact that, in response to these health and psychological issues, music students have reported seeking advice from their peers and teachers rather than from medical professionals [24]. In (medical) practice, this has added another layer of difficulty, as musicians have been known to refuse treatment for depression on the basis of this information, fearing that it would end their creative process [22].

Reflecting on these contradictions, we felt that the best way forward was to use qualitative tools to explore how classical musicians who have recovered from depression reflect on the experience of their illness. How did participants experience and cope with their depression (e.g. in terms of symptoms, support, therapy, problems specific to musicians / symptoms of depression and advice for music students)? The aim was to gain more in-depth knowledge as to how to better support musicians with depression, as well as the awareness and improved use of existing support structures. In view of a possible interdependence with their educational environment described above, we decided to focus on classical musicians who had already completed their studies at a music college as participants, rather than interviewing music college students. We were broadly guided by the following questions: Are there specific depression-related issues and obstacles that musicians and/or music students are particularly prone to encountering? To what extent does the study environment/institution have an impact on music students and how relevant is the belief in creativity resulting from depression? Furthermore, it would be helpful to find out whether there is a specific order of priority when music students seek help. Could it be true that their peers/teachers are the first point of contact whilst professional medical help is typically sought as a second option? Based on these findings, we produced contextualized real-world knowledge about how professional classical musicians experienced depression.

## Methods

This qualitative study followed a problem-focused approach where a relationship between interviewer and interviewee is essential to collect trustworthy data [25]. It used content analysis to explore how music college educated classical musicians who had received a formal medical diagnosis of depressive disorder and had since recovered experienced the illness. The study took advantage of the modular design from the standardized medical questionnaire with a focus on mental wellbeing, coping mechanisms and prevention strategies [26, 27].

### Procedure

This study is designed as a non-experimental, semi-structured written interview. We adapted the interview to reflect the music college environment. We focused on depression symptoms, coping mechanisms, and prevention strategies. A fundamental aspect for the collection of accurate and reliable data in this qualitative study was a reliable and trusting relationship with participants. Consequently, we invited classical musicians who had previously participated as a study feedback group in a different study on depression and music students to take part in this study [10]. From this pool of feedback volunteers, we contacted those who had previously reported having been medically diagnosed with one or more episodes of depression. Moreover, as a final criterion for inclusion, all potential participants were required to confirm that they had been in remission for a minimum of six months. In response to their concerns about disclosing their identity through the accumulation of (minor) details and the need for reflection on the questions, participants completed the questionnaire in written form. The questionnaire permitted respondents to provide open-ended responses and to answer each question in as much or as little detail as they choose. We followed a double-blind protocol in which participants were randomly assigned numbers by the software used to collect the responses (95–101). We kept the numbers suggested by the software. It was subsequently not possible to consolidate the participant responses via their email links. This study was conducted in accordance with the university recommendations and guidelines and the protocol was approved by the Ethics Committee of the Department. All participants were over eighteen years of age and gave informed consent in writing prior to the completion of the survey.

## Results

Seven participants agreed to take part: three vocalists, two keyboard players, two high string players (violin/viola). These selected participants were distinguished professionals in their respective fields, including soloists, educators, and/or coaches. All of our participants are musicians at a stage in their careers where contracts are, on average, signed five years in the future, and all have/have had very busy work lives. Their only source of income is or has been from music, either through performing, teaching and/or coaching. They were UK or EU citizens/residents and had all graduated from music colleges. They had been medically diagnosed with one or more episodes of depression in the past and were considered to have been in remission for at least six months. Participants had studied music for an average of 8.3 years, up to and including undergraduate and postgraduate degrees. After graduation, they had worked as a professional musician or as teacher/coach for a mean of 7.3 years (ranging from a few months to 21 years, SD = 12.8). The age range was from 25 to 65 years.

They had experienced one or more depressive episodes either during their studies at college and/or during their professional careers.

**Fig. 1:**
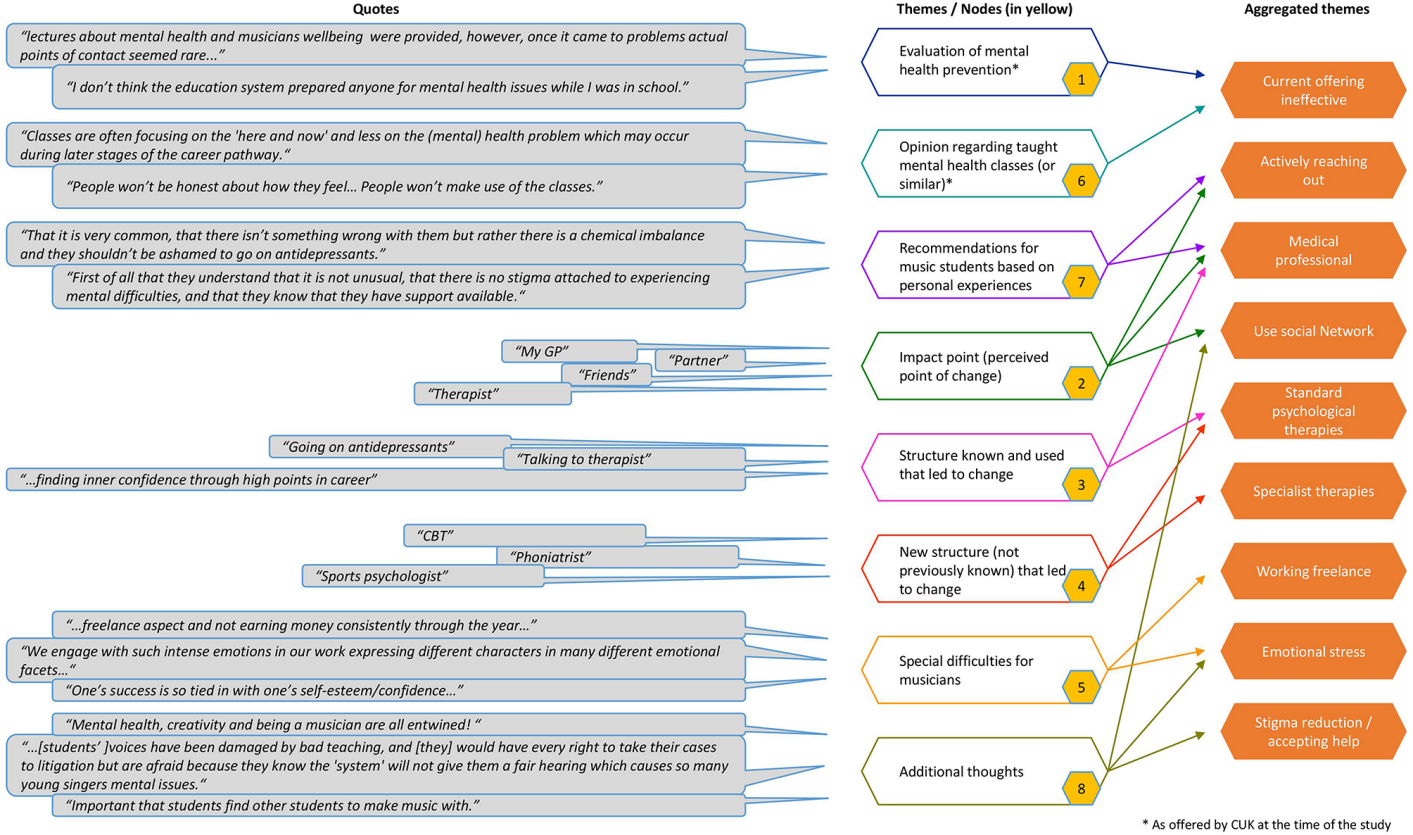
Data structure containing an overview of codes and themes with (selected) quotes

The results section is divided into two parts: the first one reflects the individual questions, and the second one analyzed the responses, using NVivo [28]. NVivo is a reliable tool for the analysis, visualization and coding of qualitative data [29]. The responses were divided into eight points, or nodes, each containing a central theme that helped to illuminate the research questions (see Fig. 1). The questionnaire consisted of four sections: (1) questions on demographics and biographical data, (2) questions on depression (e.g. symptoms, approach, diagnosis), (3) coping with depression (e.g. support, difficulties for musicians), life as a musician with depression (e.g. impact on life) (4) prevention/treatment (e.g. helpful structures, turning points, education etc.) and a section on random thoughts. While all participants responded to the biographical questions, not all questions were answered in great detail and no participant chose to respond to the section which asked about depressive symptoms. The questions that were the subject of in-depth responses are detailed below.

### Who do you turn to for support?

All participants turned to their “partner”, “family” and health care professionals such as general practitioner (“GP”, (participant 95 & 100)), “psychologist or psychiatrist” (participant 99) or a “sports psychologist” (participant 101).

### Which structure(s) did you find helpful? What were the turning points?

Participants’ responses can be divided into three sections.

1. What they did themselves or for themselves (i.e. as part of their therapy): examples included seeking “support from family and friends” (participant 101) or “learning to switch off and [be] being selfish with my spare time” (participant 96).
2. Help received from health care professionals, although it was noted that “this is not the norm”. “GP [family doctor] was very helpful and understanding” (participant 100) or “going on antidepressive” and “talking to the therapist” (participants 96 & 97) in order to regain “the confidence through high points in [their] career”.
3. Musician specific responses: A participant dealing with “pre-concert or pre-audition nerves” worked with a sports psychologist to learn the tools to fight “all forms of stage fright” (participant 101).

### In retrospect, what kind of structures would you have found helpful (that didn’t exist or weren’t available to you at the time)?

The age of the participants made a difference in responses to this question. Older participants (> 50 years) noted that there were “no structures available in place” and “we were on our own in dealing [with] whatever came up to us”. The younger participants would have appreciated access to “more information and support on wellbeing earlier on” such as “CBT”, a specialist voice therapist (“phoniatrist”) or [individual] sessions on stage fright with a psychologist.

### Based on your experience, how much would you say your education prepared you for mental health challenges?

Irrespective of the participants’ age, they concluded that their training at music college did not prepare them to deal with their depression. Although “lectures about mental health and musicians’ wellbeing were provided, however, once it came to problems actual point of contacts seemed rare” (participant 99). One participant noted that “advice from older musicians who have addressed physical issues would have been helpful” (participant 98). A participant who lost a friend to suicide recently while this friend was studying at a music college at their time of death noted that “I am acutely aware that there is still a lack of support in acute need” (participant 100). The only difference was for the participant who also teaches in the USA:

“I found that when I was in school, people didn’t talk a lot about their mental health. Now, students talk about their mental health all the time, with professors, fellow students, etc.” (participant 101).

### Based on your experience, what kinds of questions or difficulties do you foresee if or when mental health classes are taught in music colleges?

Participants all concluded that students “won’t be honest”, “may be afraid to admit they struggle to cope with emotional & mental demands” and therefore “not make use of classes” (participant 96, 97). One participant replied that “…it would be a shame to introduce the idea of suffering to students who would otherwise manage any struggle independently […]” (participant 100). Some participants elaborated that the reasons were “endemic problems” within the establishment and that “students are afraid […] it affects their grades etc.” Problems would “need to be addressed within the establishment. Only then can the concern of students [… be addressed and they] feel safe to take their concerns to those who will listen and respond” (participant 100).

### Based on your experience, what would you like current music students to know about mental health and depression?

All participants felt that current students need to know that depression “is very common” and that “support is available”; more specifically, “peer support can be there as well as professional support“. “There isn’t something wrong with them but rather there is a chemical imbalance, and they shouldn’t be ashamed to go on antidepressants“ or “have CBT” They also wanted students to learn “allow oneself help, it is also important to evaluate whether the help is professional (and actually helpful)”.

### What difficulties, if any, did you encounter as a musician, that you suspect might not be difficulties for non-musicians?

Many of the points made here could be applied to working as a freelance musician. These included, for example, living with constant (financial) stress or the need to appear successful in order to build a career. The financial aspects weighed more heavily on participants at the beginning of their careers than on those with more established careers. The need to be successful was described as universal, regardless of career stage. The freelance aspect and not earning money consistently throughout the year created a “creative drain on the brain” which added to the stress of performing and people’s expectations”. (Participant 96).

In terms of problems specific to musicians, singers noted that general stress manifested itself in physical problems such as nerves (auditions) and/or loss of voice, while instrumentalists noted a lack of confidence and/or creativity and a consequent lack of professional success. “Psychological distress […] manifested [itself] in my voice, with losing total voice strength in particularly distressful times” (participant 99). One respondent characterized the creative world of music as unique in that it inevitably involves the need to express a wide range of emotions on an almost daily basis. “Because we engage with such intense emotions in our work, expressing different characters in many different emotional facets, it is almost inevitable that we as musicians are more in touch with the extreme highs and lows.” (participant 100).

### Time for random thoughts – is there anything you would like to say before finishing this questionnaire, relating to music students’ / musicians’ mental health, that you feel hasn’t been covered?

Participants’ responses can be summarized into four categories: (a) knowledge or belief systems about mental health in the world of musicians, (b) actively using more ideas/techniques from sports psychology within the curriculum, (c) networking with other musicians, and (d) overcoming particular challenges within the music college environment. Participant 96 stated that “mental health, creativity and being a musician are all intertwined!”. Participant 98 pointed to the need to create smaller networks through “making music together”, fearing that soloists would perceive (mental) health problems more strongly if they did not have a similarly strong network. Participant 100 brought the conversation back to the music college environment and raised two different (sub)points. These were:

A) **Students’ stress related to student-teacher relationship**: for example, a desire to change teacher. The student/teacher relationship did not develop as expected by the student and the student would have preferred a different approach/teaching method. This may be because the student(s) feel that they are not playing/singing the right repertoire or are not being given enough opportunities to play recitals or auditions. These issues are difficult to assess at the beginning of a student-teacher relationship in a music college, especially when a student comes from a different cultural background than the teacher and/or the institution.

“… As someone who sees students ‘in secret’ […] because they cannot face the systemic fallout from requesting a change of teacher, I see the huge stress this puts on them. This is immoral and should be stopped.…”.

B) **Feeling of being left alone/guilt**: Music college is a special environment and as a result students feel abandoned if they do not conform and have few with few resources to address their problems.

“It became quite clear in my investigations, that sexism, racism and misogyny were rife at [….] It is particular in its ethos, as it behaves very much like an ‘old boys club’ and without doubt they close ranks when there is anything which threatens them.… promises were made about processes being put in place to ensure that there was a safe mechanism for students from other cultures to discuss their [problems]… singers whose voices have been damaged by bad teaching, and would have every right to take their cases to litigation as a result, but are afraid to do so, because they know the ‘system’ will not give them a fair hearing which causes so many young singers mental issues…”.

By analyzing all responses, regardless of the research question, we identified 9 aggregated themes. These are represented in the graph below (Fig. 1): *current offering* of help in music college *is not effective*, all participants *reached out actively*, used a *medical professional*,* made use of* their *social network*, *used standard psychological therapies*, saw the need for more *specialized therapies*, depression affected their *freelance work*. They found current students subject to *emotional stress and* more work needs to be done on *stigma reduction and accepting help*.

## Discussion

This qualitative investigation set out to gather contextual real-world knowledge about how professional classical musicians, who had been formally diagnosed with one or more episodes of depression, dealt with their condition. The objective was to gain a more profound understanding of the particular difficulties encountered by musicians with depression, with a view to developing more effective strategies for supporting these individuals, raising awareness of the existing resources available, optimizing the utilization of existing support structures and establishing the foundations for the creation of new ones. The results helped to clarify a number of initial questions, such as the way in which (medical) help was sought, the therapeutic approach used, and how depression (symptoms) affected the participants, particularly as freelance musicians.

We found that all of the participants had a strong social network of family and friends who had encouraged them to seek (medical) help. Furthermore, all participants underwent a standard course of treatment for depression, comprising cognitive-behavioral therapy and, for some, medication. Almost all participants reported that they first turned to their partner, family and/or friends before consulting. One participant felt that disclosing their mental health problems to their family and friends was the key turning point, even more so than seeking professional medical help. These responses not only confirmed earlier findings of a solid social network in this group, but also highlighted that in a crisis, musicians/music students are able to reach out beyond their peers or teachers to seek medical help if needed [10, 24]. As a result of their initial disclosure in their personal network, all participants had a subsequent consultation with their primary care physician or family physician or directly with a psychiatrist.

Given the particular belief systems about depression and the possibility that depression could be a professional liability for a musician, confiding in friends about mental health problems would speak to a solid and trusted network. Intact social networks are one of the factors that have been previously recognized as acting as a buffer against depression [30, 31]. They help to prevent rumi­nation, but they also provide informational and emotional support and the opportunity to offload the burden asso­ciated with depression [32, 33]. It also means that despite the many hours of daily practice there was sufficient time to cultivate such friendships. Participants emphasized the need for “music students to find other students to make music together”. Although participants talked about a beneficial aspect of “making music together”, our data do not allow us to extrapolate that music therapy or listening to music could be a valuable therapeutic approach for professional classical musicians. Participants estimated that soloists, who did not have the possibility to “work constantly in duos or ensembles” would consequently “have a harder time” when they encountered (mental health) problems.

In our efforts to identify depression-specific issues that affect musicians, all participants emphasized the financial stress of not being able to work, or not working as much as needed. They found that “the freelance aspect and not earning money consistently through the year” was a particular difficulty to musicians. To put this more bluntly, participants did not suffer from mood disturbances, their depression was at times so severe that they had to turn down work. They mentioned that they found it “harder to perform”, but did not say whether their depression affected their performance or other daily functioning. In retrospect, participants felt that “basic financial advice [during their time in music college] and a good sense of reality” would have helped to overcome some of this pressure. In combination with financial pressure, participants also mentioned the “creative drain on the brain, performing and stress, expectations from people”. We would argue that these points are also common to all freelance professions, and, looking at the wealth of literature on creativity in scientific research, creativity is not exclusively required in the arts alone. Furthermore, stress, pre-audition nerves or stage fright are also known by public speakers or (elite) athletes. However, there were a few issues that were unique to musicians. These were the psychological distress in form of a (complete) voice loss and the need to engage with intense emotions “on a daily basis”. What may sound less significant to a non-musician, can be existential for instance for a singer who is suddenly unable to perform or a musician who feels constantly overwhelmed and drained by emotions during practice and performance. From our participants’ responses, we cannot draw any more conclusions about anxiety, self-esteem, or whether their depression had any effect on their performance anxiety. This could be addressed in future research.

In this context, participants noted that “understanding and help” from the medical professional was “not the norm” and that they were “lucky” to have found an understanding doctor. In retrospect, participants commented that knowing more about medical specifics would have helped them find specialized help faster (e.g. access to a phoniatrist). None of the participants mentioned (chronic) pain (while playing/practicing) or the highly competitive environment of competing for the same job as vulnerable points for their depression. The only time a more competitive environment was mentioned was the belief that there was a possibility that soloists would be more affected because they would be more isolated, less able to connect with other musicians by “making music together”.

The structures provided to the participants during their studies at music college, as well as the resources made available to the next generation of students, were deemed to be ineffective in addressing mental health concerns. The issue appeared to be systemic rather than confined to the individual level, affecting organizations as a whole. In hindsight, they perceived a necessity to align themselves with an uncritical acceptance and unquestionable commitment to core values of the music college/musical world such as making sacrifices, enduring pain and/or overcoming obstacles [34, 35]. It appears evident that the participants perceived it necessary to create distance between themselves and the teaching staff at the music college, due to an existing power imbalance. The reality is that students rely on the college for guidance and support as they navigate their academic and professional musical journeys. Their final grades and professional recommendations have a significant impact on their future career prospects. This explains the participants’ observations regarding a reluctance to be open about their feelings and hesitation about disclosing their mental health struggles or to challenge the authority figures within academic institutions out of fear of jeopardizing their academic standing.

Participants who have attempted to challenge the organizational structure to ensure help for students in need of mental health support, have reported that organizations are “particular in their ethos, behaving much like “an ‘old boys club’” and without doubt they close ranks when there is anything which threatens them”. This also raises the question of whether music colleges would be an appropriate setting for the delivery of mental health education. The importance of a positive approach to performance anxiety and the need for safe spaces for mental health has long been emphasized in sport psychology [36]. Successful prevention programs have established a secure environment by delegating the program to external entities. While it is possible to set up successful in-house programs, our participants’ responses did not suggest this approach. The implementation of successful programs for music students has demonstrated significant improvements in overall wellbeing, reduction of chronic pain, and performance anxiety [37, 38]. This was accomplished by establishing a secure environment for students through the outsourcing of the program, which facilitated open communication. It changed the (perceived) power structure or the need to conform to dominant values (e.g. the necessity to make sacrifices for success as a musician).

As a final point, the culture surrounding music that rewards the experience of depression as a means of achieving a unique creative peak was briefly addressed. Participants raised this point independently and unprompted, in the section that collected their own ideas.

They felt that “creativity and mental health are entwined”. For belief systems as this, that have been established over a long time, a scientific foundation is not a prerequisite for success. It is therefore noteworthy that participants wanted future professional classical musicians to understand that “it is a chemical imbalance and there they shouldn’t be ashamed to go on antidepressants”. Participants also emphasized the fact that students “understand that it is not unusual, that there is no stigma attached to experiencing mental difficulties, and that they know that they have support available”.

Although depression is a topic that has been extensively researched in the context of musicians’ health, there is a lack of qualitative studies in this area. Furthermore, there is a noticeable absence of studies that have interviewed musicians who had been medically diagnosed with depression. Nevertheless, it is imperative to utilize the medically operationalized term “depression” in order to establish a common baseline across studies and avoid potential confusion with terms such as “poor mental health”, which encompass a broader range of concepts. Moreover, the culture surrounding music seems to encourage the suffering from depression as a means to enhance creativity and thus success. A robust social network facilitated participants’ ability to seek medical assistance and follow standard treatment protocols. The financial impact of the inability to work, decline in creative output, heightened anxiety, and difficulties engaging with emotions were significant challenges. Solutions were largely based on individual resourcefulness and not a direct result of their formal education. These novel findings indicate a lack of safe space for vulnerable music students, a decline in creativity during a depression episode and an inability to engage with emotions.

Some limitations should be noted. Although the sample was in line with qualitative studies investigating similar concepts such as grief in depression and chronic pain, it was modest [39, 40]. The study allowed us to take a more in-depth look at musicians who had suffered from depression, the generalizability of our results is limited. While our sample was able to seek help, there will be musicians who are not, who do not have a strong network of family and friends, or who refuse treatment. The number of people in the general population who seek medical assistance for depression and adhere to conventional therapeutic approaches for this condition is relatively low [41]. It would therefore be erroneous to assume that classical musicians in general are any different in this regard. Furthermore, we were constrained in our ability to provide a more detailed explanation by the fact that participants were only comfortable with a written version of the questionnaire. A face-to-face interview may have offered the chance to gain further insight or clarification. While the participants felt comfortable discussing most of their illness in anonymity, none of them have spoken publicly about their struggles with depression. In accordance with our participants’ agreement on the protection of their anonymity, we did not provide a demographic table as they were afraid that the sum of all the small details would reveal their identity. The refusal by all participants to talk about their own symptoms on the record made it impossible to delve deeper into music-specific problems. It remains questionable whether this refusal was to protect their anonymity or an inability to remember the symptoms and their effects. Further research is required to ascertain the underlying causes of this seemingly lifelong reluctance (at least many decades) in order to develop more effective tools and solutions for providing safe spaces and discussion forums for musicians in need.

We conclude that personal resourcefulness, whether of professional musicians or music college students, should not distract from the larger problem with, and suffering from, depressive disorders in this group. Whilst there is a degree of personal responsibility for one’s own healthcare, foreseeable vulnerable groups such as music college students should be offered as a matter of course additional support and empowerment. The application of evidence-based concepts from sports psychology, such as the creation of safe spaces for students or investment in better, more accessible multidisciplinary treatment, would be desirable. In this context, the momentum towards the creation of a recognized, university-based special field in musicians’ medicine, similar to sports medicine, is a welcome initiative [42, 43]. This could provide solutions to both musicians’ physical and mental health challenges, and ensure long-term career prospects and wellbeing.

## Electronic supplementary material

Below is the link to the electronic supplementary material.

Supplementary Material 1

## Data Availability

No datasets were generated or analysed during the current study.
