# Supplementary material for: The patient musician: a qualitative investigation of professional classical musicians who previously suffered from depression
Source: BMC Public Health. 2025 May 1;25:1612. doi: 10.1186/s12889-025-22656-w (PMC12044710; doi:10.1186/s12889-025-22656-w)
Supplement: Supplementary file 1 — Supplementary Material 1 [file 12889_2025_22656_MOESM1_ESM.doc]

**Questionnaire – The patient musician.**

**Demographics**

- What is your current primary instrument?
  - How were you first introduced to this instrument? Why did it appeal to you?
- How many years have you trained to be a musician?
  - Different schools? Different teachers?
- How many years have you been working freelance, on probation and/or “fest”[[1]](#footnote-2)?
  - Favourite performance events? / people to work with?

**1. Depression diagnosis**

- Roughly for how long would you say that you have suffered from depressive symptoms altogether?
  - To what extent would you say you suffered from symptoms for instance…
  - …lack of motivation?
  - …memory problems
  - …anxiety problems (if performance anxiety, significantly more than before)
  - …feeling of not being quite oneself
  - …more pain than before
  - …different sleeping pattern
  - …other
- Whom did you first approach to discuss your symptoms?
  - professional help
  - self-test
  - internet
  - friends
- At what point, if at all, did you receive a formal diagnosis? By whom? (GP, specialist, psychiatrist, other)?

**2. Coping with depression**

- Who did you turn to for support once you were sure of your diagnosis?
- What difficulties, if any, would you say that you as a musician encountered that you suspect might not be difficulties for non-musicians?
- To what extent did you try to keep your symptoms to yourself / from friends / colleagues
  - For what reason?

**3. Life as a musician with depression**

- How did your depression impact your social life, if at all?
- Which steps did you undertake to try to get better? Which of them worked for you and would you be able to say why?
  - Self-help groups
  - Trying to structure the day
  - Sport/workout/going for walks/physical activities
  - Changing diet
  - Talking to friends/family/colleagues
  - Changing/adjusting life goals
  - Faith/prayer/meditation

**4. Prevention**

1. Personal

- What kinds of strategies did/do you use to minimise the chance of a relapse?
- What structures did you find helpful? Any turning points?
- In retrospect, what kind of structures would you have found helpful (that didn’t exist or weren’t available to you at the time)?

1. The profession

- Based on your experience…
  - … how much would say your education prepared you for mental health challenges?
  - …what kinds of questions or difficulties do you foresee if or when mental health classes are taught in music colleges or music faculties?
  - …what would you like current music students to know about mental health and depression?

**5. Closing**

- Time for some random thoughts – is there anything you would like to say at this final point, relating to the issues that we have raised in our conversation today, that you feel hasn’t been mentioned before?

1. “Fest” or “fest angestellt” – German for having a contract in the DACH region for at least 12 months, usually with an orchestra or opera house. In contrast to freelance work, where a fee is paid according to the number of performances, musicians on a fest-contract are almost comparable to office workers. They benefit from regular monthly salaries and a fixed number of working hours. Even though there are variations and loopholes for employers, this type of contract gives musicians benefits that their freelance counterparts don’t have, e.g. sick leave or holiday pay while still receiving their regular salaries. [↑](#footnote-ref-2)
